# New insights of minimum requirement on legumes (*Fabaceae sp*.) daily intake in Malaysia

**DOI:** 10.1186/s40795-022-00649-x

**Published:** 2023-01-09

**Authors:** Mohd Hasni Jaafar, Noor Hassim Ismail, Rosnah Ismail, Zaleha Md Isa, Azmi Mohd Tamil, Mazapuspavina Md Yasin, Nafiza Mat Nasir, Nurul Hafiza Ab Razak, Najihah Zainol Abidin, Mahshid Dehghan, Khairul Hazdi Yusof

**Affiliations:** 1 Department of Community Health, Faculty of Medicine, UKM Medical Centre, Universiti Kebangsaan Malaysia, Cheras, Kuala Lumpur Malaysia; 2 Department of Primary Care Medicine, Faculty of Medicine, Universiti Teknologi MARA Sungai Buloh, Selangor, Malaysia; 3 Department of Diagnostic & Allied Health Science, Faculty of Health and Life Sciences, Management & Science University, Shah Alam, Selangor Malaysia; 4 Population Health Research Institute (PHRI), Hamilton Health Sciences and McMaster University, Hamilton, ON Canada

**Keywords:** Legumes, CVD, BMI, Obesity, Adults, Intake

## Abstract

**Background:**

Despite their low energy density and high nutrient content, legumes may be under-recognised as a beneficial food for the prevention and management of obesity and related diseases, such as cardiovascular disease (CVD). This study aims to analyse the moderation effect of legume intake on the relationship between BMI and the prevalence of CVD among the Malaysian adult population.

**Methods:**

This study addressed communities of urban and rural Malaysia, including adults aged between 35 and 70 years old at the baseline recruitment. A series of standardised questionnaires were used to assess legume intake, history of comorbidities and socio-demographic information. Resting blood pressure measurements and physical examinations were performed to collect blood pressure and anthropometric data. Bivariate analysis was completed to determine the association between legume intake, socio-demographic characteristics and CVD prevalence. Moderation analysis was used to quantify the moderation effect of minimum daily legume intake on the relationship between BMI and CVD prevalence.

**Results:**

This study found that those who consume less than 3 servings of legumes per day benefit from protective effects against CVD risk (POR = 0.56, 95% CI = 0.37 – 0.85). Moderation analysis of a minimum of three servings/day for the relationship between BMI and CVD prevalence showed significant effects. The group that benefited the most from this effect was those with a BMI in the range of 26 to 34 kg/m^2^.

**Conclusions:**

This study provides new insights into the recommendation for legume intake according to the relationship between BMI and the prevalence of CVD in Malaysian adults. This study recommends that those with a BMI of 26 to 34 kg/m^2^ should consume at least 3 servings of legumes per day to reduce the risk of CVD. Further prospective research is warranted to affirm these findings throughout the Malaysian population.

## Introduction

Legumes represent a polyphenol-rich category of food with beneficial associations with obesity, cardiovascular disease and other non-communicable diseases (NDC) [1]. Legumes feature properties such as low-energy–density (ED), and they are high in complex carbohydrate, protein, fibre and low glycaemic index (GI) [2]. Furthermore, they are dense in nutrients and rich in phytochemicals such as riboflavin, thiamine, folic acid, phenolic acids and flavonoids [2]. In Malaysia, the consumption of legumes is highly recommended by the Malaysian Dietary Guideline (MDG). Although at least one serving per day is recommended by the guideline [3], a previous study showed that the minimum intake is unmet [4]. The requirement of one serving of legumes can easily be achieved by the intake of one cup of dhal, two pieces of tempeh or tofu or 1.5 cups of baked beans [3].

Previous studies have shown that legume consumption is beneficial for the prevention and management of obesity, cardiovascular disease (CVD) and diabetes [5–7]. Legume intake has also been found to be inversely associated with non-cardiovascular causes of death and overall total mortality. This is due to polyphenols that contains in legumes such as phenolic acid and flavonoids, which are known to alleviate CVD [8]. These benefits are optimal when three to four servings of legumes are consumed per day (equivalent to 375–500 g/day) [9]. According to the National Health and Morbidity Survey (NHMS), the prevalence of obesity increased from 17.7% in the year 2015 to 19.7% in the year 2019 [10]; at 30.2%, the rate of obesity was particularly high among older Malaysian individuals [11]. Therefore, mitigating obesity is a principal factor in the reduction of CVD risk [12]. Previous literature suggests that optimal dietary patterns should emphasise the intake of fruits, vegetables (not including potatoes), plant-based fats and proteins, beans, legumes, grains and nuts as carbohydrate sources [13]. However, despite the uniquely high nutrient density and low ED of legumes, they are often overlooked for the prevention and management of obesity and other related diseases.

Despite the recommendations of the Malaysian government to consume legumes as part of daily dietary intake, studies of the relationship between legume intake, obesity and CVD are limited in Malaysia. Thus, this study aims to investigate the moderation effect of legume intake on the relationship between BMI and the prevalence of CVD among the Malaysian adult population.

## Methodology

### Study design and population

PURE is a large-scale international study on the incidence, mortality and risk factors associated with non-communicable diseases among individuals from urban and rural communities in 21 countries, including Malaysia. Coordinated by the Population Health Research Institute (PHRI) in Hamilton, Ontario, Canada, data collection for the study began in 2007, and follow-up is projected to continue until 2030. In Malaysia, data collection is done collaboratively by the Universiti Kebangsaan Malaysia (UKM) and the Universiti Teknologi MARA (UiTM). This study has enrolled 15,792 Malaysian individuals aged between 35 and 70 years old at the baseline phase. The design of the PURE study has been described in previous studies [14–17]. Assessment for all the component of this paper was carried out as part of PURE study in Malaysia.

Participants were conveniently recruited from selected urban and rural areas throughout peninsular and east Malaysia. After the field researcher acquired permission from the community leaders, health screening and promotion booths were set up in the communities’ assembly halls. With the help of community leaders, residents were informed and invited to visit the booths. Interested and eligible participants were briefed about the study. Once written informed consent were obtained, medical histories were taken, basic physical examinations were conducted and home visit were set. During the home visits, other individuals living in the same household were asked to join the study. Only the household members intend to continue living in their current home for a further 4 years were selected to join this study to ensure the feasibility of long-term follow-up. All participants provided written informed consent after they understood that their participation was entirely voluntary.

To ensure standardised methods of data collection, research assistants were trained with comprehensive operation manuals, videos and workshops. Data were transferred electronically to the project office and the coordinating centre at PHRI for quality control. The protocol was approved by the Hamilton Health Sciences Research Ethics Board (PHRI), the Research and Ethics Committee (UKM Medical Centre) and the Research Ethics Committee (UiTM) (Project code: PHUM-2012–01).

### Measurements

Participants were classified as part of the CVD group if they self-reported of having been diagnosed by certified medical practitioner with coronary heart disease (CHD), heart failure, stroke hypertension or recorded high blood pressure (SBP/DBP > 140/90) [9]. Two recordings of blood pressure after 5 min of rest in a sitting position with the use of an automatic Omron blood pressure monitor (model HEM-7111).

Long term dietary intake of participants was measure using validated food frequency questionnaire [9]. Participants were asked “during the past year, on average, how often have you consumed the following foods or drinks” and the list of food items was given. The frequencies of consumption varied from never to more than 6 times /day. Standard serving sizes (e.g. an egg) were assigned to each food item. To compute the daily food and nutrient intakes, the reported frequency of consumption for each food item was converted to daily intake and then was multiplied by the portion size. Legumes included long beans, winged beans, peas and soybean products (tofu). Legume intake was reported as servings per day (1 serving = 1 cup = 72 g) [18]. Participants were grouped based on their intake into < 3 and ≥ 3 servings of legumes per day. 

Information on demographic characteristics was obtained from the validated Adult’s PURE questionnaire [15, 16, 19]. Demographic characteristics included age (rounded to the nearest year), sex, race (Malay or non-Malay), marital status (single, married or divorced), education level (none, primary, secondary or tertiary) and employment status (yes or no). The residency area (urban or rural) of the participants was defined based on local government gazetted area. Urban areas were defined as areas occupied by more than 150 residents per square kilometre. Height and weight were measured using a portable stature meter and the TANITA (BC-558 Ironman®) segmental body composition analyser. Height was measured to the nearest 1 cm and body weight was measured to the nearest 100 g, when participants wore no shoes and only light clothing. Body mass index (BMI) was derived by dividing weight by height squared, and individuals were categorized as obese (≥ 30 kg/m^2^) or non-obese (< 30 kg/m^2^).

### Statistical analysis

The data were analysed using the SPSS version 26. The chi-square test was used to assess differences among the CVD group according to the following variables: legume intake, BMI, age, gender, race, marital status, education level, employment status and residency. Then, a simple logistic regression was used to determine the association between individual factors and CVD status. Biologically plausible characteristics with a p-value < 0.3 at simple logistic regression were included in the multiple logistic regression model, for which the prevalence of rejecting the null hypothesis was set at 0.05. Confounding factors of BMI, age, gender, marital status, education level, employment status and residency were included in the final logistic regression model. The results were reported as frequencies, percentages, prevalence odds ratios (POR) and 95% confidence intervals (95% CI).

Moderation analysis was employed to examine the effects of legume intake (moderator) on the relationship between BMI and CVD prevalence [20]. The moderation analysis replicated the Dawson method using a two-way interaction equation as shown below [21]. Only curvilinear interactions are shown in the results as these supersede the linear interaction analysis.

$$Y={b}_{0}+{b}_{1}X+{b}_{2}{X}^{2}+{b}_{3}Z+{b}_{4}XZ+{b}_{5}{X}^{2}Z+\varepsilon$$

(Two-way interaction equation)

where:

Y, dependent variable = CVD.

X, independent variable = BMI.

Z, moderator = legume intake.

In this analysis, age, residency (urban/rural), education level and employment status were included as controlled variables. These variables were selected based on a *p* < 0.05 as determined by the previous simple logistic regression and conform to known individual and social determinants of health. CVD incidence and legume intake were measured as categorical variables, while age and BMI were measured as continuous variables. Both age and BMI were Z-standardised before moderation analysis. Then, moderation effects were visualised graphically using the online Microsoft Excel spreadsheet by Dawson [21]. According to Dawson, the moderation effect was considered significant when both of the following conditions were met: (1) the interaction between the dependent and independent variables had a *p* of less than 0.05 as determined by the moderation analysis and (2) the visualisation of the interaction exhibited an intercept with the moderator’s slope [21].

## Results

A total of 15,366 participants provided a complete personal medical history of CVD. Among these, 9,384 participants completed the FFQs regarding legume intake without missing data on age, gender or BMI. The prevalence of CVD in this population was 44.97%. The mean (± SD) of BMI and age of this study population was 27.48 (± 5.04) kg/m^2^ and 54.80 (± 8.76) years old respectively among those with CVD (Table 1). Those with CVD were more common among obese (62.3%), aged 61 to 70 years old (65.7%) and those without formal education (57.4%).

**Table 1:** Characteristics of study population and regression analysis between demographic characteristic and legumes intake with CVD status

	CVD status, mean (± SD)			
Demographic	Yes	No		
BMI	27.48 (5.04)	25.95 (4.28)		
Age	54.80 (8.76)	48.64 (9.07)		
	CVD status, frequency (%)			
	Yes	No	*p* value	POR (95% CI)
Demographic				
BMI			< 0.001*	
Obese	947 (62.3)	572 (37.7)		**2.32 (2.07 – 2.60)**
Non-obese	3273 (41.6)	4592 (58.4)		Ref
Age			< 0.001*	
35–40	273 (19.2)	1152 (80.8)		Ref
41–50	1085 (35.4)	1980 (64.6)		**2.31 (1.99 – 2.69)**
51–60	1619 (53.9)	1384 (46.1)		**4.94 (4.25 – 5.74)**
61–70	1243 (65.7)	648 (34.3)		**8.09 (6.88 – 9.52)**
Gender			0.077	
Male	1824 (44.0)	2326 (56.0)		Ref
Female	2396 (45.8)	2838 (54.2)		1.07 (0.99 – 1.17)
Race			0.269	
Malay	3679 (45.2)	4457 (54.8)		1.07 (0.95 – 1.21)
Non- Malay	520 (43.5)	675 (56.5)		Ref
Education			< 0.001*	
None	545 (57.4)	405 (42.6)		**2.72 (2.27 – 3.26)**
Primary	1696 (55.6)	1352 (44.4)		**2.54 (2.19 – 2.93)**
Secondary	1613 (37.7)	2671 (62.3)		**1.22 (1.06 – 1.41)**
Tertiary	363 (33.1)	734 (66.9)		Ref
Employment Status			< 0.001*	
Yes	1918 (39.7)	2918 (60.3)		Ref
No	2284 (50.7)	2225 (49.3)		**1.56 (1.44 – 1.69)**
Marital status			< 0.001*	
Single	68 (31.6)	147 (68.4)		Ref
Married	3759 (44.4)	4699 (55.6)		**1.73 (1.29 – 2.31)**
Separated	388 (55.3)	313 (44.7)		**2.68 (1.94 – 3.70)**
Residency			< 0.001*	
Urban	1814 (40.4)	2677 (59.6)		Ref
Rural	2406 (49.2)	2487 (50.8)		**1.43 (1.32 – 1.55)**
Legumes intake				
Minimum 3 servings/day			0.006*	
< 3	4165 (44.8)	5126 (55.2)		**0.56 (0.37 – 0.85)**
≥ 3	55 (59.1)	38 (40.9)		Ref

^*^significant at *p* < 0.05

The simple logistic regression showed that significant factors associated with CVD in this study were BMI (POR = 2.32, 95% CI = 2.07 – 2.60), increased age [41–50 (POR = 2.31, 95% CI = 1.99 – 2.69), 51–60 (POR = 4.94, 95% CI = 4.25 – 5.74) and 61–70 (POR = 8.09, 95% CI = 6.88 – 9.52)], lower education level [without education (POR = 2.72, 95% CI = 2.27 – 3.26), primary education (POR = 2.54, 95% CI = 2.19 – 2.93) and secondary education (POR = 1.22, 95% CI = 1.06 – 1.41)], lack of employment (POR = 1.56, 95% CI = 1.44 – 1.69), marital status [married (POR = 1.73, 95% CI = 1.29 – 2.31) and separated (POR = 2.68, 95% CI = 1.94 – 3.70)] and lastly, rural residents (POR = 1.43, 95% CI = 1.32 – 1.55). This study demonstrated that the consumption of less than 3 servings of legumes per day appears to have a protective effect against CVD (POR = 0.56, 95% CI = 0.37 – 0.85).

**Fig. 1:**
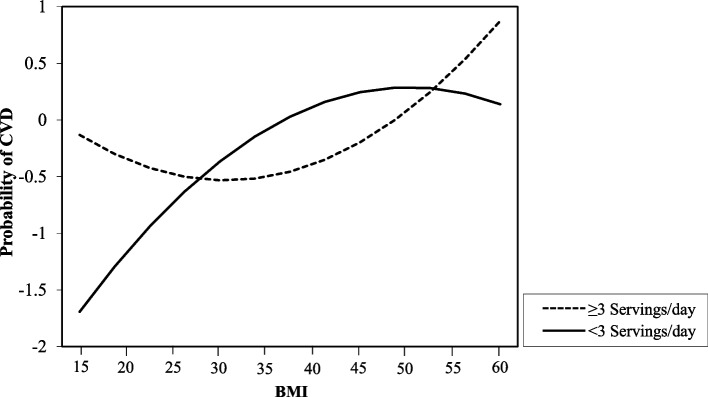
Moderating effect of legume intake (< 3 & ≥ 3 servings/day) on the curvilinear BMI-prevalence of CVD relationship (two-way interaction)

The moderation analysis demonstrated that a minimum of three servings per day had significant effects on the relationship between BMI and CVD prevalence (Table 2). The analysis of the moderation effect of frequency of consumption on BMI and the risk of CVD indicated that the consumption of a minimum of three daily servings had significant effects (p = 0.045) compared to less than three servings per day. This result demonstrates that the relationship between BMI and the prevalence of CVD is far more curvilinear in nature for individuals with higher legume intake (the dotted line) than for those with lower intake autonomy (the solid line) (Fig. 1). Based on the vertex of the dotted curvilinear graph, the magnitude of the effect of the number of servings appears to be especially promising for those with a BMI between 26 and 34 kg/m^2^.

**Table 2:** Moderation analysis of legumes intake on BMI-prevalence of CVD relationship

Minimum 3 servings/day	B	S.E	*p* value
Intercept	-0.226	0.330	0.245
BMI	0.477	0.028	< 0.001
BMI^2^	-0.030	0.013	0.014
Legume	-0.216	0.310	0.243
BMI x Legume	-0.141	0.278	0.307
**BMI**^**2**^** x Legume**	**0.397**	**0.239**	0.045*

^*^significant at *p* < 0.05

## Discussion

This study demonstrates that the moderating effect of legumes on the relationship between BMI and CVD prevalence is significant for three servings per day (216 g) among those with a BMI between 26–34 kg/m^2^. Nonetheless, a higher BMI may negate the protective benefits of legumes against CVD [22, 23]. Evidence suggests that to significantly lower risk of major CVD or CVD mortality, at least 4–5 servings of legumes (around 500 g) should be consumed per day [24]. One systematic review and meta-analysis addressing the intake of nuts and legumes and the prevention of ischemic heart disease, stroke, and diabetes found that 400 g per weekly 100-g provided significant benefits [25].

Several studies have suggested that the consumption of legumes could enhance weight management [26], an effect that may be attributed to their low GI, and high protein and fibre content. According to Maphosa and Jideani, the low GI carbohydrate in legumes stabilises blood sugar and insulin levels, helping the consumer feel satiated for longer periods of time [26]. Furthermore, consumption of low energy-dense foods such as legumes induces satiety at a lower energy intake [2]. The interaction of protein, amylose starch and fibre content may greatly lower the rate of digestibility, thereby increasing energy expenditure associated with digestion [2]. These factors could contribute to dietary patterns of eating smaller quantities at a reduced frequency, which is ideal for weight management.

Based on the results of this study, those with a BMI in the range of 26–34 kg/m^2^ should be advised to consume at least 3 servings of legumes per day to reap the beneficial effects and reduce their risk of CVD. A handful of epidemiological and clinical studies have linked legume consumption with reduce serum total cholesterol (TC), LDL-C, and TAG levels and increase high-density-lipoprotein-cholesterol (HDL-C) levels [27, 28]. Legumes, which are high in bean protein and water-soluble fiber, that may reduce blood levels of triglycerides and cholesterol, thus lowering the risk of CVD [29, 30]. Apart from that, legumes which are rich in potassium, magnesium, and dietary fiber shown a positive impact on blood pressure management. In eight trials involving > 500 people, half of whom were overweight or obese, reductions in blood pressure (systolic and mean arterial blood pressure) have been seen in subjects that had eaten legumes [6].

Limitations to this study include its use of cross-sectional baseline data, wherein the exposure and outcome were simultaneously assessed. There is therefore insufficient evidence of a temporal relationship between legume intake, BMI and the prevalence of CVD. Another limitation of this study was only four types of legumes were included. This may be explaining the extreme small number of respondents taking 3 servings of legumes per day (1% of the study population). Thus, support for the recommended level of legume intake indicated in this study requires further research, such as considering all types of legumes available in Malaysia and controlled trials study design, to confirm the optimal intake of legumes to reduce the prevalence of CVD. Besides, this study does not capture the effect of total calorie intake but only focused on legumes intake alone. Thus, future studies should investigate both total calorie intake together with legumes intake. Future studies should also consider addressing an adequately ethnically diverse sample in order to accurately represent the general Malaysian population. Further studies of the usefulness of legumes for weight management represent a worthwhile avenue for future research, especially since this food is readily available and widely consumed in the daily diets of the Malaysian population.

## Conclusion

This study demonstrated that, when the direct relationship between BMI and CVD prevalence is considered, the Malaysian population benefits from the consumption of at least 3 servings of legumes per day to lower the risk of CVD especially those with a BMI in range of 26 to 34 kg/m^2^. Legumes represent a valuable food that should be emphasised in weight management programs administered by health practitioners, especially because the food is widely available in Malaysia.

## Data Availability

The data that support the findings of this study are available from PHRI but restrictions apply to the availability of these data, which were used under license for the current study, and so are not publicly available. Data are however available from the corresponding author upon reasonable request and with permission of PHRI.

## Abbreviations

CVD: Cardiovascular diseases
BMI: Body mass Index
ED: Energy Density
MDG: Malaysia Dietary Guideline
NHMS: National Health and Morbidity Survey
PURE: Prospective Urban and Rural Epidemiological Study
FFQs: Food frequency questionnaires
